# Analyzing Comprehensive QoS with Security Constraints for Services Composition Applications in Wireless Sensor Networks

**DOI:** 10.3390/s141222706

**Published:** 2014-12-01

**Authors:** Naixue Xiong, Zhao Wu, Yannong Huang, Degang Xu

**Affiliations:** 1 School of Mathematics and Computer Science, Hubei University of Arts and Science, Xiangyang 441053, China; E-Mails: nxiong@coloradotech.edu (N.X.); yannonghuang@gmail.com (Y.H.); pcxinx@163.com (D.X.); 2 School of Computer Science, Colorado Technical University, Colorado Springs, CO 80907, USA

**Keywords:** wireless sensor networks, quality of service, security constraint, services composition, universal generating function

## Abstract

Services composition is fundamental to software development in multi-service wireless sensor networks (WSNs). The quality of service (QoS) of services composition applications (SCAs) are confronted with severe challenges due to the open, dynamic, and complex natures of WSNs. Most previous research separated various QoS indices into different fields and studied them individually due to the computational complexity. This approach ignores the mutual influence between these QoS indices, and leads to a non-comprehensive and inaccurate analysis result. The universal generating function (UGF) shows the speediness and precision in QoS analysis. However, only one QoS index at a time can be analyzed by the classic UGF. In order to efficiently analyze the comprehensive QoS of SCAs, this paper proposes an improved UGF technique—vector universal generating function (VUGF)—which considers the relationship between multiple QoS indices, including security, and can simultaneously analyze multiple QoS indices. The numerical examples demonstrate that it can be used for the evaluation of the comprehensive QoS of SCAs subjected to the security constraint in WSNs. Therefore, it can be effectively applied to the optimal design of multi-service WSNs.

## 1. Introduction

In recent years, WSNs have evolved with new features such as large scale, high device heterogeneity and the capability of supporting multiple applications [1]. WSNs are optimized for low-power, low-cost and a small form factor. Enterprise-IT systems are typically equipped with orders of magnitude more resources [2]. In Enterprise-IT it is important to adapt business processes and the underlying software infrastructure quickly and flexibly to react to changes on the markets [3]. To achieve this goal, some organizations focus on modeling, analysis and adaptation of business processes since the early 1990s [4]. Yet, while Service-Oriented Architecture (SOA) is prospering in Enterprise-IT, WSNs have—despite contrary prognoses—not yet found their way into enterprises. In recent years, some approaches have been presented for the seamless integration of WSNs with the existing, widely deployed SOA technologies such as XML, Web Services, and the Business Process Execution Language to build a service application based on wireless sensor networks [5].

In parallel with this development, WSNs are envisioned to become an integrated part of the Future Internet where they extend the Internet to the physical world [6]. Under the combined efforts of WSNs and Internet, these two trends lay the groundwork for a new class of applications where all kinds of devices ranging from simple sensor nodes (SNs) to large-scale application servers interact to drive business processes not possible before. In this way data stemmed from a WSN may influence the control flow of a business process in real-time or even trigger a business process [7]. To achieve this level of integration, WSNs must seamlessly interoperate with existing widely deployed SOA technologies such as XML, Web Services, and the Business Process Execution Language to name only a few [8]. In recent years, some approaches have been presented for the seamless integration of WSNs with these SOA technologies to successfully build a service application based on wireless sensor networks [9–11]. In these approaches, WSNs are packaged as some standard Web services which can be published, located, and invoked across the Web. Therefore, based on Business Process Execution Language (BPEL), these WSNs services can be combined into a workflow to fulfill some tasks by the way of services composition [12–14].

The SCAs can be achieved in a centralized or distributed fashion. For simplicity, this paper uses a centralized fashion. The WSNs Service Broker (WSB) deployed in the management server can act as a central decision node receiving service requests from users and dispatching the service tasks to the suitable SNs.

**Figure 1.**
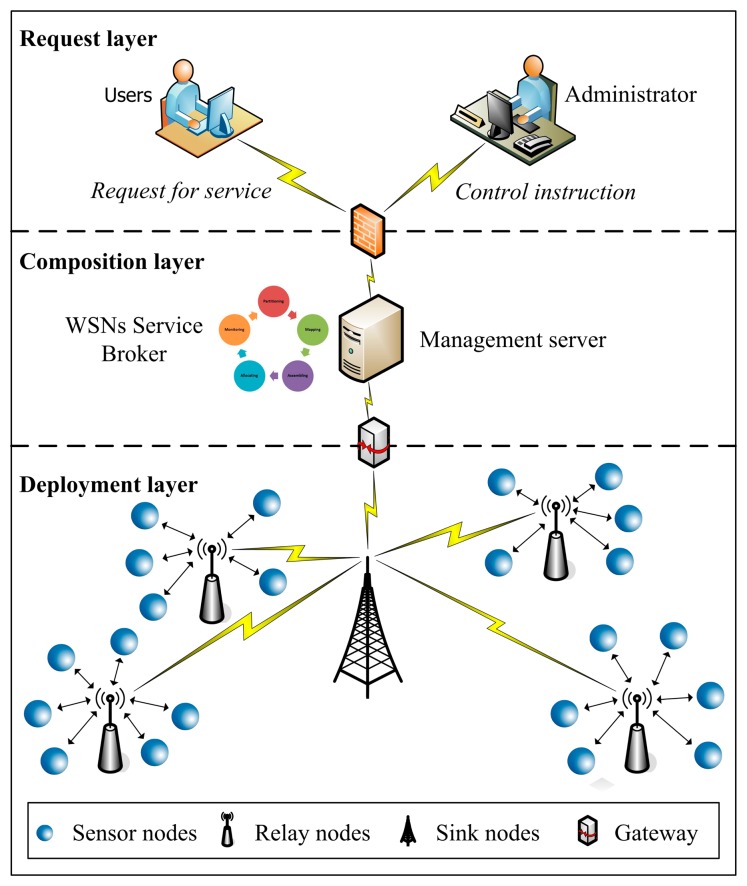
Architecture of SCAs in WSNs.

The SCAs in WSNs can be approximated as a three-tier architecture as shown in Figure 1, which is composed of the request layer, the composition layer and the deployment layer. The top layer represents the service requests from users, as well as the control instructions from system administrators. These service requests and control instructions are submitted to the composition layer through a firewall which improves the access security of the SCAs in WSNs by separating the application server from the users and the administrators.

The middle layer represents the logic entity of the SCAs in WSNs. As the resource management center, the WSB runs in the management server of WSNs. The WSB is responsible for the receiving of service request from the top layer, the partitioning of service task, the mapping from sub-tasks to atom-services, the assembling of an application of the services composition, and the allocating of SNs in the bottom layer for an SCA, as well as the operation monitoring of WSNs.

As a core component of WSNs service system, the WSB manages users' service request and controls the startup, access to, and sharing of data resources from the bottom layer. When a user's service request is received, the WSB partitions the service task into some sub-tasks according to adomain-specific business rules at first. Each sub-task represents a certain specific business operation. Then the WSB maps these sub-tasks into an atom-service which can be fulfilled by a single SNs according to the functionality of each atom-service. The service task can be fulfilled by the cooperation between these atom-services. Thus, under the coordination by the WSB these atom-services form a services composition. When all computing tasks are fulfilled by SNs, the WSB is responsible for receiving the observed data from the bottom layer through a gateway and sending them back to the users through the firewall.

The bottom layer represents the physical entity of the SCAs in WSNs. In the bottom layer, some SNs are deployed in different locations. These SNs make up a network through the wireless connections by the relay nodes and sink nodes. The relay nodes are responsible for the data transmission between the sink nodes and SNs located in a geographic range. The sink nodes are responsible for the data transmission between the WSB and all of the SNs in the WSNs through the different relay nodes.

From the aspect of system architecture, the application of services composition in WSNs is a type of Internet components based on WSNs services, which is built by services composition technique [15]. As a type of abstract of distributed software system running on the Internet which is open, dynamic and difficult to be controlled, there are many differences between the Internet components and the traditional software system, such as structure, operation mechanism, correctness guarantees, development method and life cycle [16]. Being different from the traditional software model, the WSNs services, as a type of Internet components, exist in each SN on the WSNs service platform with a proactive software service form [17].

In the framework of WSNs service system, the WSNs services composition fulfills the users' service request through the collaboration among the SNs. Typically, the execution route and the selection for SNs are dynamically determined according to the operating condition during the execution of an SCA [18]. In addition, the outside SNs can be dynamically added in a WSNs service system at any time. And it can be selected to execute during the execution of an SCA by the late binding technology. Therefore, there is no knowing all the SNs, as well as their operating condition and performance indices before the end of the operation of an SCA, which are the essential differences with the traditional software. The QoS assurance methods of the traditional software are mostly based on a software model constant during the execution [19–21]. Thus, they are not suitable for the SCA which is based on services composition techniques.

On the other hand, the computational burden is the crucial factor in solving optimization problems where the QoS indices, such as cost, execution time, credibility and reputation, have to be evaluated for a great number of possible solutions along the search process [22]. The traditional QoS assessment methods, such as Boolean Models [23], Markov Process [24] and Monte-Carlo simulation technique [25], have some disadvantages, either the applicability of only small-scale system or too much time consumed for executing model [26].

Unlike the traditional software QoS assurance technique, the QoS assurance method for the application of services composition in WSNs pays more attention to the mechanism of flexible QoS measures, deduction and adoption based on cumulative evaluation of operation information in an open running environment [27]. Therefore, the fast QoS optimization methods for the application of services composition in WSNs have great theoretical research value.

In order to provide better QoS to users, SCAs must have more adaptability to collect various changes in real-time, and to adjust online according to pre-established strategies at runtime [28]. However, with the closed, controllable and static user's requirement in the background, the traditional software QoS optimization methods lack the ability to dynamically adapt themselves to the changes both in running environment and user's requirement [29,30]. Therefore, they cannot be employed in QoS optimization for the application of services composition in WSNs, so the fast QoS prediction method for SCAs has an important realistic requirement.

At present, researches on QoS optimization for SCAs is still just starting. Due to the open and dynamic running environment, continuously variable user's requirement, randomly selected SNs and the own characteristics of loose coupling and long transaction, the QoS optimization for the application of services composition in WSNs are confronted with severe challenges, which seriously restrict the further development, application and extension of the SCAs [31]. Faced with the urgent demands for SCAs with high-reliability and high-performance from the government, economy and commerce fields such as e-government, e-commerce and e-banking, fast QoS optimization is the key to promote the successful development, application and extension of the SCAs [32], which provides a flexible and effective mechanism of QoS measure for SCAs by deduction and adoption based on the summative evaluation of operating information.

In recent years, many previous studies have focused on the security guarantees at the network layer in WSNs [33,34]. However, it is impossible to assure the overall security of SCAs in WSNs only depending on the security guarantees of the network layer [35–37]. Therefore, the security must be considered together with other QoS indices in analyzing comprehensive QoS of SCAs in WSNs.

Therefore, this paper focuses on the comprehensive evaluation of multiple QoS indices including security for SCAs in WSNs, which aims at providing a selection criterion for some potential solutions for designers of SCAs. On this basis, this paper further focuses on analyzing the contribution of each SN to the total evaluation, which aims at providing some potential optimization probabilities to the designers. The current solutions cannot solve the above problems well. Most current researchers separate performance, reliability, and security into different fields and study them individually. However, in fact, performance, reliability and security are closely related and affect each other, in particular when the WSA is implemented [38–41]. For example, when a task is divided into different ASs simultaneously executed by SNs, the performance is high, but the reliability can be low because failure of any SN will make the entire task incomplete. This causes the task to restart, which inversely increases its execution time (*i.e.*, reduces its performance). Therefore, it is worth having some redundant SNs to execute the same AS, especially for those failure-prone SNs. However, too many redundancies, even though improving the reliability, can decrease the performance by not fully parallelizing the execution of different AS and considerably reduces data security as multiple replicas of the same data are processed by different SNs, which increases the chances of unauthorized access. For different types of users (or different usage scenarios), the security requirements may be different, which relates to the security levels of data accessed by users. That is to say, the security of service applications based on wireless sensor networks is interconnected with the security levels of the accessed data. In the design of the service application system, the high security level should be used for the data with high confidentiality to prevent the occurrence of unsafe events. For example, the probability of the occurrence of unauthorized access can be reduced by limiting the number of redundant key nodes. Obviously, doing so may reduce the system performance and/or reliability. Therefore, ensuring absolute security is unrealistic in the design of a service application system. Designers usually have to compromise between the security, the performance and the reliability. Thus, performance, reliability and security should be studied together in WSN service analysis.

In order to assure the lowest security strength of SCAs in WSNs satisfying users' individualized security requirements and improve other QoS indices as much as possible, this paper studies the analysis method for the comprehensive QoS with security constraints of SCAs in WSNs based on the VUGF technology. Our contributions are as follows: (I) We present the working mechanisms of SCAs in WSNs and analyze the necessity of comprehensive assessment on multiple QoS indices; (II) We analyze the disadvantages of the classic UGF, and present a VUGF technique that can obviously improve the efficiency on comprehensive QoS assessment in a parallel computing environment; (III) Employing our VUGF, we present a composition calculation method of multiple QoS indices for SCAs in WSNs, which can work out the total comprehensive QoS assessment by using a fast algebraic procedure; (IV) We present an identification method for the key SNs based on analyzing the contribution of each SN to the total evaluation, which can most likely improve the total comprehensive QoS of SCAs in WSNs. This method provides an approach for designers to further optimize the total comprehensive QoS.

The remainder of this paper is organized as follows: Section 2 gives the working mechanisms of SCAs in WSNs. Section 3 presents the VUGF technique as well as comparisons with the classic UGF. Following this, the approaches of VUGF-based composition calculation of multiple QoS indices are proposed in Section 4. On this basis, Section 5 presents the analysis methods of multiple QoS indices for SCAs in WSNs in detail. In order to illustrate our approach, some numerical examples and analysis process are described in Section 6. Finally, the conclusions and future work are given in Section 7.

## 2. Working Mechanisms of WSNs Service Broker

**Figure 2.**
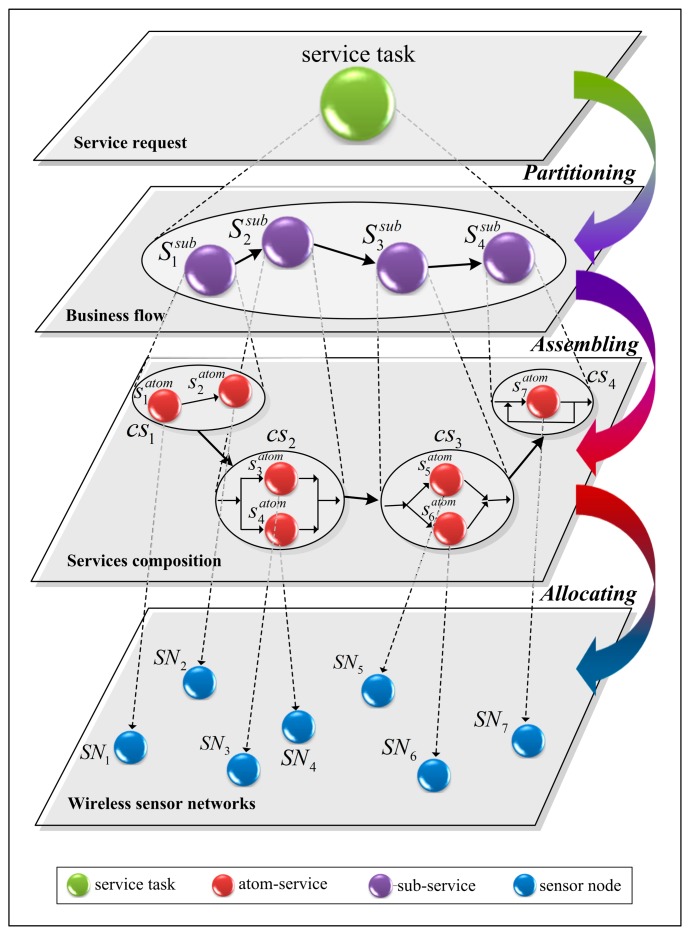
Working mechanisms of the WSN service broker.

Under the service oriented architecture, the independent Web services are built for every SN in WSNs, which are deployed in the management server beforehand. The functionalities of each SN, such as initialization, starting and accessing data, are encapsulated in some methods in the corresponding Web service. Therefore, these Web services can be regarded as the agents of those SNs. By invoking the appropriate methods in the Web service using the SOAP message protocol, the expected functional effects provided by the corresponding SNs can be obtained. From the outside of the WSNs service system, the WSB can be regarded as the agent of users who made service requests. Under the control of WSB, the service requests from users are fulfilled. The control process includes some operations, such as aportioning the service task, assembling the composite service, and allocating SNs. The working mechanisms of WSB are shown in Figure 2.

When receiving a service request from a user, a suitable workflow process will be chosen by the WSB according to some related domain-specific business rules, which are depicted as the business flow layer in Figure 2. These workflow processes are physically implemented as a group of workflow templates. Each template is composed of some interconnected predefined sub-services which are denoted as $S_{i}^{\text{sub}}$. It is noteworthy that these sub-services are the abstract definitions of some larger-granularity services in the business layer rather than some physical Web services in the operation layer. Therefore, the determination of workflow process by the WSB is actually the partition of service tasks, which is depicted as the evolution process from the service request layer to the business flow layer in Figure 2.

The Web service is a self-defined, self-describing, modular application that can be published, located, and invoked across the Internet based on UDDI/OWL-S. After choosing a suitable workflow process for a user's service request, the WSB can discover and match the required Web services deployed physically in the WSNs service system for each sub-service within the workflow. In most cases, the accomplishment of the functionalities of a sub-service needs the cooperation of some Web services, named atom-service and denoted as $S_{i}^{\text{atom}}$ in Figure 2, corresponding to the same SN or different SNs.

These interlinked atom-services, which cooperate to fulfill the service task specified by a sub-service, form a services composition which is denoted as *CS_k_* in Figure 2. To improve the efficiency, the domain expert and the system builders usually define the mapping relationships between the sub-services and the Web services beforehand.

Corresponding to each sub-service within the workflow, all of the atom-services are assembled as a composite service which can be invoked by the WSB to fulfill the user's service request. Therefore, the discovery and matching of the required atom-services by the WSB is actually the services composition assembly process, which is depicted as the evolution from the business flow layer to the services composition layer in Figure 2.

After the composite service is assembled, the WSB can dynamically deploy each atom-service to some suitable SN according to its execution order defined in the composite service. Normally, the system builders can deploy some redundant SNs to improve system's reliability and scalability. Each SN has a unique address which can be used by the WSB for the configuration of every atom-service. Therefore, the WSB can dynamically select the most appropriate SNs for each atom-service according to their functionality and non-functional indices, which is depicted as the evolution from the services composition layer to the wireless sensor networks layer in Figure 2. To reduce the time consumed for dynamic configuration, we deploy and configure an atom-service for each SN beforehand. The dynamic QoS indices of each SN are recorded by the WSB. Therefore, based on these atom-services deployed for every SN the selection of the appropriate SNs is translated into the selection of the appropriate atom-services.

The selection of atom-services for an SCA is crucial to its QoS. The QoS analysis of the alternative solutions for an SCA should be performed in order to find the best solution. In this paper, we choose the UGF technique to build our QoS analysis method. The next section introduces the classical UGF technique first, and then presents an improved UGF technique (VUGF) which can be used for analyzing multiple QoS indices simultaneously.

## 3. VUGF Technique

In order to better introduce our VUGF, the classic UGF technique is introduced first. On this basis, we analyze its disadvantages and then we put forward our innovative thoughts and give the definition of our VUGF. Finally, we compare the classic UGF and our VUGF.

### 3.1. Classical UGF Technique

Some systems can perform their tasks with various distinguished levels of efficiency usually referred to as performance rates. A system that can have a finite number of performance rates is called a Multi-State System (MSS) [42]. Any system consisting of different units that have a cumulative effect on the entire system performance has to be considered as a MSS [43].

Since the QoS indices of atom-services can take different values, the SCA should be considered as a MSS with different QoS levels depending on different combination of available and failed SNs with different performance, reliability, security and creditability. MSS was introduced in the mid-1970s in [44,45]. In these works, the basic concepts of MSS were primarily formulated, the system structure function was defined, and its properties were initially studied. The notions of minimal cut set and minimal path set were introduced in the MSS context, as well as the notions of coherence and element relevancy.

MSS QoS analysis relates to systems for which one cannot formulate an “all or nothing” type of failure criterion. Such systems are able to perform their task with partial performance (intensity of the task accomplishment). Failures of some system elements lead only to the degradation of the system performance.

The SCA is a typical MSS which has a variety of operation states (or failure states) besides the normal operation states and complete failure states. In other words the SCA can runs on multiple QoS levels. The failure or QoS degradation of some SNs will cause QoS degradation of the entire SCA. Thereby, the whole SCA presents multiple QoS levels.

The QoS analysis method based on MSS theory can broadly define the QoS indices of each atom-service (represents corresponding SN) and the entire SCA in detail. It can also analyze the effect of QoS variation of each atom-service (or SN) on QoS of the entire SCA thoroughly, as well as the gradual process of SCA failure.

Generally, the approaches of MSS QoS analysis can be divided into four different types: (1) an extension of the Boolean models to the multi-valued case; (2) the stochastic process (mainly Markov and semi-Markov) approach; (3) the Monte-Carlo simulation technique; (4) the u-function approach.

The approach based on the multi-valued Boolean models is historically the first method that was developed and applied for MSS QoS evaluation. It is based on the natural expansion of the Boolean methods to Multi-State systems.

The stochastic process methods that are widely used for the MSS QoS analysis are more universal. These methods can be applied only to relatively small MSS because the number of system states increases dramatically with the increase in the number of system elements.

Even though almost every real world MSS can be represented by a Monte-Carlo simulation for QoS assessment, the main disadvantages of this approach are the time and expense involved in the development and execution of the model.

Thus, the first three approaches have some disadvantages, either the applicability of only small-scale MSS or too much time consumed for executing the model. The computational burden is the crucial factor when one solves optimization problems where the QoS indices have to be evaluated for a great number of possible solutions along the search process. This makes the use of the first three approaches in QoS optimization problematic [46].

On the contrary, the UGF technique is fast enough, and can be applied in the QoS analysis of a large-scale MSS [47]. This is because that UGF technique allows one to find the performance distribution of the entire MSS based on the performance distributions of its elements by using a fast algebraic procedure. For the above reasons, we chose the UGF technique as a fundamental one to explore an approach for simultaneously analyzing multiple QoS indices of an SCA. In the next section, we present an enhanced UGF technique, named Vector UGF (VUGF).

### 3.2. Vector UGF Technique

Although the classic UGF has some outstanding advantages, such as its speediness and precision, its framework only supports analyzing a single variable, *i.e.*, a single QoS index, in an algebraic procedure. In other words, it cannot analyze multiple QoS indices simultaneously, which exposes its disadvantage of low efficiency.

In addition, the classic UGF can only express one QoS index in an UGF expression by a pair of the possible value variable and the probability variable. Based on this UGF expression, there is only one QoS index can be computed in an algebraic procedure. In other words, each QoS index is computed separately in classic UGF. Therefore, the classic UGF cannot depict the relationships between the QoS indices, and it does not consider the QoS constraints in the analysis procedure. Different QoS indices may affect each other. For example, some SNs can fail when running the AS, so the execution time is also affected by the SN reliability. Similarly, the communication links in SNs can fail during the data transmission. Thus, the communication reliability influences the service time as well as data transmission speed in the communication channels, so the analysis results worked out by the classic UGF are incomplete and inaccurate.

To overcome these disadvantages of the classic UGF, we present an improved UGF, named VUGF, to study the simultaneous analysis of multiple QoS indices for an SCA in an algebraic procedure. The VUGF inherits the outstanding advantages that allow one to find the entire MSS performance distribution based on the performance distribution of its elements by using a fast algebraic procedure. An analyst can use the same recursive procedures with a different physical nature of QoS and different types of element interaction. The VUGF is defined as follows:

It is assumed that ***G*** = {*G*_1_, *G*_2_, …, *G_m_*} is an *m*-dimensional discrete random vector, where *G*_1_, *G*_2_, …, *G_m_* represent *m* different QoS indices of an atom-service *G* in the SCA, such as cost, execution time, security, credibility, and reputation. The probability distribution of ***G*** can be described as two vector sets ***g*** and ***q***.

The vector ***g*** represents the *M* possible values of the vector ***G***, that is the atom-service *G* have *M* different states corresponding to these *m* different QoS indices *G*_1_, *G*_2_, … *G_m_*. The ***g*** can be expressed by the following formula:

(1) $$g=\{g_{1},g_{2},\cdots ,g_{M}\}$$

where:

(2) $$g_{l}=\{g_{1,1},g_{1,2},\cdots ,g_{1,m}\},l=1,2,\ldots ,M$$

In other words, *g_1_*_,1_, *g_1_*_,2_, …, *g_1,m_* are the values of QoS indices *G*_1_, *G*_2_, …, *G_m_*, respectively.

The vector ***q*** represents the probability that the QoS indices *G*_1_, *G*_2_, …, *G_m_* take the values *g_1_*_,1_, *g_1_*_,2_, …, *g_1,m_*. The ***q*** can be expressed by the following formula:

(3) $$q=\{q_{1},q_{2},\cdots ,q_{M}\}$$

where:

(4) $$q_{\text{l}}=\mathrm{Pr}\{G=g_{l}\}=\mathrm{Pr}\{G_{1}=g_{1,1},\cdots ,G_{m}=g_{1,m}\}$$

where:

(5) $$\sum_{\text{l}=1}^{M}q_{l}=1$$

Thereby, the VUGF of vector ***G*** can be defined as:

(6) $$U_{\text{G}}(z)=\sum_{\text{l}=1}^{M}q_{l}z^{{\text{g}}_{l}}$$

**Figure 3.**
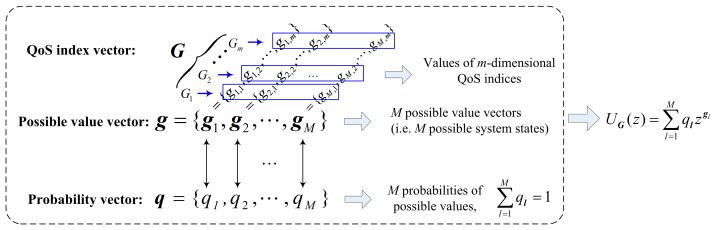
Relationships between ***G***, ***g*** and ***q*** in the VUGF.

Figure 3 describes the relationships between the QoS index of vector ***G***, the possible value vector ***g*** and the probability vector ***q*** in the VUGF.

**Figure 4.**
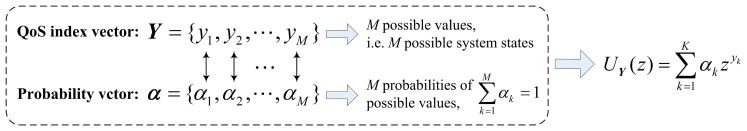
Relationships between ***Y*** and **α** in the UGF.

Figure 4 describes the relationships between the QoS index vector ***Y*** and the probability vector **α** in the classic UGF. Comparing the definitions of the VUGF and the classic UGF in Figures 3 and 4, the biggest difference is that the ***g****_l_* in Formula (6) is a vector, while the *g_l_* is a variable in the definition of the classic UGF. The vector ***g****_l_* enables that the possible values of multiple QoS indices are expressed in a VUGF formula simultaneously, which supports the synchronous computation of multiple QoS indices. Employing our VUGF, the WSNs service broker can use a cluster to parallel work out the estimate for each QoS index. For example, each estimation task of QoS indices can be assigned to some different parallel computing units (workers). Our VUGF program can these estimation tasks in parallel based on MATLAB^®^ Distributed Computing Server (MDCS (The MathWorks, Inc., Natick, MA, USA)) and Parallel Computing Toolbox (PCT). Therefore, compared with the classic UGF, our VUGF can provide better performance in parallel computation conditions.

Being different from the traditional UGF, VUGF can combine the security and multiple different types of QoS indices into a unified, parallel computation-enabled framework by converting the variables of QoS indices and their probability distributions into the corresponding vectors. Thus, analyzing comprehensive QoS with security constraints can be fulfilled. The benefit to do so are not only that the computational efficiency can be greatly improved by the parallel computation in computing models, but also a reasonable compromise between the security, the performance and the reliability can be obtained easily for the optimal system design. The next section describes the mechanism of composition calculation for the multiple QoS indices from the single atom-services to the entire SCA based on VUGF.

## 4. Composition Calculation of Multiple QoS Indices Based on VUGF

### 4.1. Definition of Composition Operator

The VUGF of the entire SCA can be derived from the VUGFs of all atom-services within this SCA by the composition calculation. In order to facilitate the description of the composition calculation, we define a composition operator Ω as follows.

Define *N* discrete random vectors ***G***_1_, ***G***_2_, …, ***G****_N_*, and their VUGFs are *U*_1_(*z*), *U*_2_(*z*), …, *U_N_*(*z*) respectively. Thus, the VUGF of a vector function ***f***(***G***_1_, ***G***_2_, …, ***G****_N_*) is the composition calculation of *U*_1_(*z*), *U*_2_(*z*), …, *U_N_*(*z*) which can be expressed as the following formula:

(7) $$U(z)=\Omega (U_{1}(z),U_{2}(z),\cdots ,U_{N}(z))$$

When calculating Formula (7), the following VUGF's characteristics will be applied:

(8) $$\Omega (U_{1}(z),\cdots ,U_{k}(z),U_{k+1}(z),\cdots ,U_{N}(z))=\Omega (U_{1}(z),\cdots ,U_{k+1}(z),U_{k}(z),\cdots ,U_{N}(z))$$

(9) $$\Omega (U_{1}(z),\cdots ,U_{k}(z),U_{k+1}(z),\cdots ,U_{N}(z))=\Omega (\Omega (U_{1}(z),\cdots ,U_{k}(z)),\Omega (U_{k+1}(z),\cdots ,U_{N}(z)))$$

By applying the characteristics mentioned above, the obtained VUGF of the vector function ***f***(***G***_1_, ***G***_2_, …, ***G****_N_*) takes the following forms:

(10) $$U(z)=\sum_{s=1}^{M_{\text{sys}}}q_{s}\cdot z^{{\text{x}}_{s}}$$

where the *M_sys_* is the number of the possible values (*i.e.*, possible system states) of the vector function ***f***(***G***_1_, ***G***_2_, …, ***G****_N_*), and $M_{\text{sys}}\leq \prod_{i=1}^{N}M_{i}$ owing to the collection of like terms; the ***x****_s_* is the vector which composes of the possible values of the vector function ***f***(***G***_1_, ***G***_2_, …, ***G****_N_*); the *q_s_* is the probability of ***x****_s_*.

### 4.2. Composition Calculation for the VUGF of Two Random Vectors

Define an *m*-dimensional discrete random vector ***H*** = {*H*_1_, *H*_2_, …, *H_m_*}. *H*_1_, *H*_2_, …, *H_m_* that represents *m* different QoS indices of another atom-service *H* within the SCA, respectively. The probability distribution of *H*_1_, *H*_2_, …, *H_m_* can be described by two vector sets ***h*** and ***p***. The ***h*** = {*h*_1_, *h*_2_, …, *h_M′_*}, ***h****_k_* = {*h_k_*_,1_, *h_k_*_,2_, …, *h_k,m_*} (1 ≤ *k* ≤ *M′*), express all possible *M′* values which ***H*** can take. ***p*** = {*p*_1_, *p*_2_, …, *p_M′_*} express the probabilities that ***H*** takes the value of ***h****_k_*. According to Formula (6), the VUGF of ***H*** is:

(11) $$U_{H}(z)=\sum_{k=1}^{M^{\prime }}p_{k}\cdot z^{{\text{h}}_{k}}$$

Define an *m*-dimensional discrete random vector ***D*** = {*D*_1_, *D*_2_, …, *D_m_*}. It is a function of vectors ***G*** and ***H***, *i.e.*, ***D*** = ***f***(***G***, ***H***), where:

(12) $$D_{i}=f_{i}(G_{1},\cdots ,G_{m},H_{1},\cdots ,H_{m})$$

Thereby, the vector ***D*** represents the *m* different QoS indices of the composite service composing of two arbitrary atom-services *G* and *H* within the SCA. According to Equations (7)–(9), the VUGF of ***D*** can be obtained by the following composition calculation:

(13) $$U_{D}(z)=\Omega (U_{\text{G}}(z),U_{\text{H}}(z))=\sum_{l=1}^{M}\sum_{k=1}^{M^{\prime }}q_{l}\cdot p_{k}\cdot z^{\text{f}({\text{g}}_{l},{\text{h}}_{k})}$$

As seen from the above formula, *U_D_*(*z*) is composed of *M* × *M′* terms. The resulting probabilities of each term within the VUGF of ***D*** are equal to the products of the probabilities of the corresponding terms within the VUGF of ***G*** and ***H***, and the resulting values of QoS indices can be worked out according to the specification of the vector function ***f***(***g****_l_*, ***h****_k_*) where *l* and *k* are the order number of the possible values of QoS indices in ***G*** and ***H***. The vector function ***f***(***g****_l_*, ***h****_k_*) can take the following expression:

(14) $$f(g_{l},h_{k})=(f_{1}(g_{l,1},h_{k,1}),\cdots ,f_{m}(g_{l,m},h_{k,m}))$$

In the calculation process of the above formula, the alike terms in the VUGF can be collected, which can obviously simplify the calculation process. For example, if ***f***(***g***_1_, ***h***_1_) = ***f***(***g***_2_, ***h***_2_) then the two terms *q*_1_*p*_1_*z^f^*^(^*^g^*^1^,*^h^*^1)^ and *q*_2_*p*_2_*z^f^*^(^*^g^*^2^,*^h^*^2)^ can be collected into (*q*_1_*p*_1_+*q*_2_,*p*_2_)z*^f^*^(^*^g^*^1^,*^h^*^1)^, so the number of terms in the resulting VUGF *U_D_*(*z*) may be less than *M* × *M′*. Combining like terms can reduce the number of possible values of the vector ***D***, so then the calculation workload can be reduced.

For the different types of QoS indices and the different composite structures in the SCA, different computational methods should be selected to calculate each term *f_i_*(*g_l,i_*, *h_k,i_*) in the vector function ***f***(***g****_l_*, ***h****_k_*). In order to facilitate the description of composition calculation, we define several commonly used operators as follows:

(15) $$\underset{\mathrm{sum}}{⊗}(g_{l,i},h_{k,i})=g_{l,i}+h_{k,i}$$

(16) $$\underset{\max}{⊗}(g_{l,i},h_{k,i})=\max(g_{l,i},h_{k,i})$$

(17) $$\underset{\max}{⊗}(g_{l,i},h_{k,i})=\max(g_{l,i},h_{k,i})$$

(18) $$\underset{\mathrm{product}}{⊗}(g_{l,i},h_{k,i})=g_{l,i}\times h_{k,i}$$

According to the structures of the composite services within the SCA, one can obtain the values of various types of QoS indices of each composite service by applying the above operators to calculate each term *f_i_*(*g_l,i_*, *h_k,i_*) in vector function ***f***(***g****_l_*, ***h****_k_*).

For example, assume that a composite service is composed of two atom-services *G* and *H*; the discrete random vectors ***G*** and ***H*** represent the four kinds of QoS indices (*i.e.*, cost, security, execution time, and credibility) of *G* and *H*, respectively. Here execution time is used for depicting the performance of atom-service. ***G*** and ***H*** can be respectively expressed as: ***G*** = {*G_cost_*, *G_security_*, *G_exec_time_*, *G_credibility_*}, ***H*** = {*H_cost_*, *H_security_*, *H_exec_time_*, *H_credibility_*}.

The vector ***g****_l_* = {*g_l,cost_*, *g_l,security_*, *g_l,exec_time_*, *g_l,credibility_*} (1 ≤ *l* ≤ *M*) represents the *M* possible values that the vector ***G*** may take. Similarly, the discrete random vector ***h****_k_* = {*h_k,cost_*, *h_k,security_*, *h_k,exec_time_*, *h_k,credibility_*} (1 ≤ *k* ≤ *M′*) represents the *M′* possible values that the vector ***H*** may take.

Aiming at the various connections of *G* and *H* in the structure of composite service in the SCA, the different operators should be selected to calculate the values of various QoS indices of this composite service. The four common types of connections in the composite structure are discussed as follows:

(1) *G* and *H* in series connection
  ◆ Using the operator $\underset{\mathrm{sum}}{⊗}$, the values of two QoS indices “cost and execution time” of the composite service composing of *G* and *H* can be worked out according to the following two formulas, respectively:
    (19) $$f_{\text{cost}}(g_{l,\text{cost}},h_{k,\text{cost}})=\underset{\mathrm{sum}}{⊗}(g_{l,\text{cost}},h_{k,\text{cost}})=g_{l,\text{cost}}+h_{k,\text{cost}}$$
    (20) $$\begin{array}{ll} f_{\text{exec}\_\text{time}}(g_{l,\text{exec}\_\text{time}},h_{k,\text{exec}\_\text{time}}) & =\underset{\mathrm{sum}}{⊗}(g_{l,\text{exec}\_\text{time}},h_{k,\text{exec}\_\text{time}})\\ & =g_{l,\text{exec}\_\text{time}}+h_{k,\text{exec}\_\text{time}} \end{array}$$
    where *f_exec_time_*(*g_l,exec_time_,h_k,exec_time_*) is used for depicting the performance of the composite service.
  ◆ Using the operator $\underset{\mathrm{product}}{⊗}$, the values of QoS index “security” of the composite service composing of *G* and *H* can be worked out according to the following formula:
    (21) $$\begin{array}{ll} f_{\text{security}}(g_{l,\text{security}},h_{k,\text{security}}) & =\underset{\mathrm{product}}{⊗}(g_{l,\text{security}},h_{k,\text{security}})\\ & =g_{l,\text{security}}\times h_{k,\text{security}} \end{array}$$
  ◆ Using the operator $\underset{\min}{⊗}$, the values of QoS index “credibility” of the composite service composing of *G* and *H* can be worked out according to the following formula:
    (22) $$\begin{array}{ll} f_{\text{credibility}}(g_{l,\text{credibility}},h_{k,\text{credibility}}) & =\underset{\min}{⊗}(g_{l,\text{credibility}},h_{k,\text{credibility}})\\ & =\min\{g_{l,\text{credibility}},h_{k,\text{credibility}}\} \end{array}$$
(2) *G* and *H* in parallel connection
  ◆ Using the operator $\underset{\mathrm{sum}}{⊗}$, the values of QoS index “cost” of the composite service composed of *G* and *H* can be worked out according to Equation (19).
  ◆ Using the operator $\underset{\max}{⊗}$, the values of QoS index, execution time, of the composite service composing of *G* and *H* can be worked out according to the following formula:
    (23) $$\begin{array}{ll} f_{\text{exec}\_\text{time}}(g_{l,\text{exec}\_\text{time}},h_{k,\text{exec}\_\text{time}}) & =\underset{\max}{⊗}(g_{l,\text{exec}\_\text{time}},h_{k,\text{exec}\_\text{time}})\\ & =\max\{g_{l,\text{exec}\_\text{time}},h_{k,\text{exec}\_\text{time}}\} \end{array}$$
  ◆ Using the operator $\underset{\min}{⊗}$, the values of QoS index “security” of the composite service composing of *G* and *H* can be worked out according to the following formula:
    (24) $$\begin{array}{ll} f_{\text{security}}(g_{l,\text{security}},h_{k,\text{security}}) & =\underset{\min}{⊗}(g_{l,\text{security}},h_{k,\text{security}})\\ & =\min\{g_{l,\text{security}},h_{k,\text{security}}\} \end{array}$$
  ◆ Using the operator $\underset{\min}{⊗}$, the values of QoS index “credibility” of the composite service composing of *G* and *H* can be worked out according to Equation (22).
(3) *G* executed circularly *c_G_* times
  ◆ The values of QoS indices “cost and execution time” of the composite service composing of *G* executed circularly *c_G_* times can be worked out according to the following formulas, respectively:
    (25) $$f_{\text{cost}}(g_{l,\text{cost}})=c_{G}\cdot g_{l,\text{cost}}$$
    (26) $$f_{\text{exec}\_\text{time}}(g_{l,\text{exec}\_\text{time}})=c_{G}\cdot g_{l,\text{exec}\_\text{time}}$$
  ◆ The values of QoS indices “security” of the composite service composing of *G* executed circularly *c_G_* times can be worked out according to the following formula:
    (27) $$f_{\text{security}}(g_{l,\text{security}})={\left(g_{l,\text{security}}\right)}^{c_{G}}$$
  ◆ The values of QoS indices “credibility” of the composite service composing of *G* executed circularly *c_G_* times can be worked out according to the following formula:
    (28) $$f_{\text{reputation}}(g_{l,\text{reputation}})=g_{l,\text{reputation}}$$

In addition to the above situations, the atom-services *G* and *H* may also be combined in a branch structure with the selection probabilities *p_G_* and *p_H_* (*p_G_* + *p_H_* = 1), respectively. That is to say that the composite service will dynamically select either the atom-service *G* with the selection probability *p_G_*, or the atom-service *H* with the selection probability *p_H_* to execute according to the runtime conditions. In this case, the VUGF of the composite service can be obtained as follows:

(29) $$U_{D}(z)=p_{\text{G}}\cdot U_{G}(z)+p_{\text{H}}\cdot U_{H}(z)$$

### 4.3. Recursive Computation of the VUGF of Multiple Random Vectors

The VUGF of multiple random vectors represents the vector of QoS indices of a composite service composing of multiple atom-services in the SCA. It can be expressed as follows:

(30) $$\begin{array}{ll} U(z) & =\Omega (U_{{\text{G}}_{1}}(z),U_{{\text{G}}_{2}}(z),\cdots ,U_{{\text{G}}_{n}}(z))\\ & =\sum_{l_{1}=1}^{M_{1}}\sum_{l_{2}=1}^{M_{2}}\cdots \sum_{l_{n}=1}^{M_{n}}p_{l_{1}}p_{l_{2}}\cdots p_{l_{n}}\cdot z^{\text{f}\left({\text{g}}_{1},{\text{g}}_{2},\cdots ,{\text{g}}_{n}\right)} \end{array}$$

Through the following recursive computation process, the VUGF ***f***(***g***_1_, ***g***_2_, …, ***g****_n_*) of multiple random vectors can be obtained using Equations (15)–(28) according to the composite structure of all atom-services within the SCA:

(31) $$\begin{array}{ll} f(g_{1},g_{2},\cdots ,g_{n})=f(g_{1},g_{2}), & \\ f(g_{1},g_{2},\cdots ,g_{n})=f(f(g_{1},g_{2},\cdots ,g_{n}),g_{e}) & \text{for}\;e=3,\cdots ,n. \end{array}$$

## 5. Analysis of Multiple QoS Indices Including the Security

**Figure 5.**
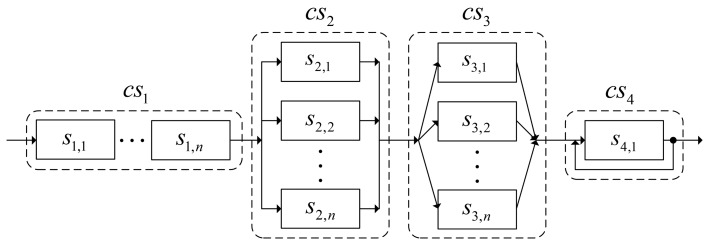
Composite structure of an example of SCA instance.

In the suggested analysis method, the security is considered as a specific QoS index standing at parity with other QoS indices. In order to facilitate the description of analyzing multiple QoS indices, a composite structure of an example instance of an SCA is shown in Figure 5.

The SCA instance is composed of four composite services: *cs*_1_, *cs*_2_, *cs*_3_, and *cs*_4_ which represent the four types of composition structures mentioned above, respectively. The composite service *cs*_1_ is composed of *n* atom-services *s*_1,1_, *s*_1,2_, …, *s*_1_,*_n_* in serial connection. The composite service *cs*_2_ is composed of *n* atom-services *s*_2,1_, *s*_2,2_, …, *s*_2_,*_n_* in parallel connection. The composite service *cs*_3_ is composed of *n* atom-services *s*_3,1_, *s*_3,2_, …, *s*_3_,*_n_* in branch connection with the selection probability *P_s_*_3,1_, *P_s_*_3,2_,…,*P_s_*_3_,*_n_* respectively. The composite service *cs*_4_ is composed of an atom-services *s*_4,1_ to be executed circularly *c_s_*_4,1_ times. In the above SCA instance, the calculation process of the VUGF of the multiple QoS indices comprises three steps. Firstly, for each atom-service, its VUGF of QoS indices is calculated. Secondly, for each composite service, its VUGF of the QoS indices is calculated based on the VUGFs of all the atom-services it contains. Thirdly, for the entire SCA instance, its VUGF of the QoS indices is calculated based on the VUGFs of all the composite services it contains. Finally, the comprehensive QoS of the entire SCA instance can be estimated. The details of each step of the above-mentioned process are described in the next sections.

### 5.1. Calculating the VUGF of QoS Indices for Each Atom-Service

Let the atom-service *s_i,j_* in the composite service *cs_i_* have *M_ij_* states. At time *t*, the QoS of all atom-services in the composite service *cs_i_* can be expressed by ***g****_ij_*(t)={***g****_ij_*_1_(*t*),***g****_ij_*_2_(*t*),…,***g****_ij_*_M_*_ij_*(*t*)}, where ***g****_ijl_*(*t*) (*l*∈[1,*M_ij_*]) is the QoS vector of the atom-service *s_i,j_* in the *l*-th state, and the corresponding state probability vector is ***q****_ij_*(*t*)={*q_ij_*_1_(*t*),*q_ij_*_2_(*t*),…,*q_ij_*_M_*_ij_*(*t*)}.

According to Equation (6), the VUGF of the atom-service *s_i,j_* can be expressed by the following formula:

(32) $$U_{ij}\left(z,t\right)=\sum_{l=1}^{M_{ij}}q_{\text{ijl}}\left(t\right)z^{{\text{g}}_{\text{ijl}}\left(t\right)}$$

Normally, the QoS ***g****_ij_*(*t*) and the corresponding state probabilities ***q****_ij_*(*t*) of each atom-service *s_i,j_* can be directly obtained through online monitoring or estimate of the operational log of each atom-service from WSB. Following this, based on the VUGFs of all the atom-services, the VUGF of the QoS indices of each composite service can be calculated, which is described in the next section.

### 5.2. Calculating the VUGF of the QoS Indices for Each Composite Service

The composite services *cs*_1_, *cs*_2_, and *cs*_3_ are all composed of *n* different atom-services in serial, parallel, and branch connection, respectively. Their QoS states can be expressed in a unified form by the following formula:

(33) $$X_{i}=f(G_{i1},G_{i2},\cdots ,G_{in})$$

where ***X****_i_*, 1 ≤ *i* ≤ 3, are the QoS vectors of the above composite services, and ***G****_i_*_1_, ***G****_i_*_2_, …, ***G****_in_* are the QoS vectors of each atom-service, respectively. The above formula is called the structure function which describes the relationships between the QoS of the composite service and the atom-services.

According to the VUGF of atom-services described by Formula (32) and the structure function described by Formula (33), the VUGF of the composite services *cs*_1_, *cs*_2_, and *cs*_3_ can be worked out using Equations (7)–(9), which can be expressed in a unified form as follows:

(34) $$U_{i}(z,t)=\Omega (U_{i1}(z,t),\cdots ,U_{in_{i}}(z,t))=\sum_{k=1}^{M_{i}}q_{ik}\left(t\right)z^{{\text{x}}_{ik}\left(t\right)}$$

where *M_i_* (1 ≤ *i* ≤ 3) is the number of the states of each composite service; {***x****_i_*_1_(t),…,***x****_iMi_*(t)} (1 ≤ *i* ≤ 3) is the QoS of each composite service at time *t*; {*q_i_*_1_(t),…,*q_iMi_*(t)} is the corresponding state probability.

Aiming at the parallel connection of atom-services, such as in the composite services *cs*_2_, in the event that a composite service has many parallel atom-services, the number of states of this composite service, for example *M*_2_, will be very large. In order to improve the computation speed of the VUGF of the entire SCA instance, a state redistricting method can be applied to reduce the number of states of the composite service, which is described in the case of the composite service *cs*_2_ as follows:

Redistrict the *M*_2_ states of *U*_2_(*z*, *t*) into *M*_2_′ states (*M*_2_′<*M*_2_). The size of *M*_2_′ can be decided by the computational accuracy and the computing speed required actually. After redistricting *M*_2_ into *M*_2_′, the QoS of the composite service *cs*_2_ will be changed as follows:

$$\begin{array}{ccc} x_{21}(t),\cdots ,x_{2c}(t) & \Rightarrow & {x^{\prime }}_{21}(t),\\ & \vdots & \\ x_{2d}(t),\cdots ,x_{2M_{2}}(t) & \Rightarrow & {x^{\prime }}_{2{M^{\prime }}_{2}}(t). \end{array}$$

The corresponding state probability will be changed as follows:

$$\begin{array}{ccc} q_{21}(t),\cdots ,q_{2c}(t) & \Rightarrow & {q^{\prime }}_{21}(t)=q_{21}(t)+\cdots +q_{2c}(t),\\ & \vdots & \\ q_{2d}(t),\cdots ,q_{2M_{2}}(t) & \Rightarrow & {q^{\prime }}_{2{M^{\prime }}_{2}}(t)=q_{2d}(t)+\cdots +q_{2M_{2}}(t). \end{array}$$

Thus, the VUGF of the composite service *cs*_2_ will be changed into:

(35) $$U_{2}\left(z,t\right)=\sum_{k=1}^{{M^{\prime }}_{2}}{q^{\prime }}_{2k}\left(t\right)z^{{x^{\prime }}_{2k}\left(t\right)}$$

The above state redistricting method can be neatly applied in the calculation process of the VUGF of the composite services or the entire SCA instance. The composite service *cs*_4_ takes a loop connection. *G* is executed circularly *c_G_* times in *cs*_4_. This can be transformed into a serial connecting format where the same *c_G_* atom-services *s*_4,1_ are executed sequentially. Thereby, the VUGF of the composite services *cs*_4_ can also be worked out according to the approaches presented above.

### 5.3. Calculating the VUGF of the QoS Indices for Entire SCA Instance

The example of an SCA instance shown in Figure 5 comprises four composite services in serial. The structure function of the SCA instance can be expressed by the following formula:

(36) $$Y=f(X_{1},X_{2},X_{3},X_{4})$$

where the ***Y*** is QoS vector of the entire SCA instance; ***X***_1_, ***X***_2_, ***X***_3_, ***X***_4_ are the QoS vectors of the composite services in the SCA instance, respectively. The VUGF of the QoS indices of the entire SCA instance can be obtained by using Equations (7)–(9) as follows:

(37) $$U(z,t)=\Omega (U_{1}(z,t),\cdots ,U_{4}(z,t))=\sum_{s=1}^{M_{s\text{y}s}}q_{s}\left(t\right)z^{y_{s}\left(t\right)}$$

where *M_sys_* is the number of states of the entire SCA instance; ***y****_s_*(*t*) = {*y*_1_(*t*), …, *y_Msys_*(*t*)} (1 ≤ *s* ≤ *M_sys_*) is the QoS indices of the entire SCA instance; the ***q****_s_*(*t*) = {*q*_1_(*t*), …, *q_Msys_*(*t*)} (1 ≤ *s* ≤ *M_sys_*) is the corresponding state probability.

### 5.4. Estimating the Comprehensive QoS of an SCA Instance

To estimate the comprehensive QoS, such as the reliability, the usability and the output-performance, of an SCA instance, a vector ***w*** = (ω_1_, ω_2_, …, ω*_m_*) is defined as the QoS constraint for the entire SCA instance from users or designers, such as the maximum cost, the minimum credibility, the maximum execution time, and the minimum security. When the QoS index of the ***y****_s_*(*t*) of the entire SCA instance meets the QoS constraint ***w*** in the state *s*, it is denoted by ***y****_s_*(*t*)∝***w*** in this paper. To make the VUGF of the QoS indices of the entire SCA instance meet the QoS constraint ***w***, the constraint operator δ*_w_* is defined as follows:

(38) $$\delta_{\text{w}}\left(q_{s}\left(t\right)z^{{\text{y}}_{s}\left(t\right)},w\right)=\{\begin{array}{cc} q_{s}\left(t\right), & y_{s}\left(t\right)\propto w,\\ 0, & \text{otherwise}. \end{array}$$

The above formula indicates that some QoS index terms, *i.e.*, some *q_s_*(*t*)*z^ys^*^(^*^t^*^)^ in the VUGF $U\left(z,t\right)=\sum_{s=1}^{M_{s\text{y}s}}q_{s}\left(t\right)z^{y_{s}\left(t\right)}$, can be omitted when they don't satisfy the constraints ***w***. Thereby, those terms will be deleted by setting *δ_w_*(*q_s_*(*t*)*z^ys^*^(^*^t^*^)^,***w***)=0.

Thereby, the VUGF of the comprehensive QoS indices of the entire SCA instance under the constraint ***w***, *i.e.*, *U_w_*(*z*, *t*), can be calculated by the following formula:

(39) $$U_{\text{w}}\left(z,t\right)=\delta_{\text{w}}(\sum_{s=1}^{M_{s\text{y}s}}q_{s}\left(t\right)z^{{\text{y}}_{s}\left(t\right)},w)=\sum_{s=1}^{M_{\text{sys}}}\delta_{\text{w}}(q_{s}\left(t\right)z^{y_{s}\left(t\right)},w)$$

The expected value of each QoS index, defined in the vector ***Y*** such as cost, execution time, security, credibility, and reputation, of the entire SCA instance can be worked out by the following formula:

(40) $$E\left({\text{y}}_{s}\left(t\right)|U_{\text{w}}\left(z,t\right)\right)=\sum_{{\text{y}}_{s}(t)\propto \text{w}}\frac{q_{s}\left(t\right)}{\sum_{y_{s}(t)\propto \text{w}}q_{s}\left(t\right)}y_{s}\left(t\right)$$

In addition, some other QoS indices of the entire SCA instance, such as reliability, usability, and output-performance, can also be worked out based on the VUGF of the multiple QoS indices of the entire SCA instance.

In order to facilitate the description, three QoS operators, *i.e.*, reliability operator δ*_R_*, usability operator δ*_A_*, and output-performance operator δ*_G_*, are defined. The approaches of estimating the reliability, usability, and output-performance of the entire SCA instance are described as follows: to begin with, the reliability operator δ*_R_* is defined. The reliability of the entire SCA instance in time *t* under the constraint ***w*** can be calculated by the following formula:

(41) $$R\left(t\right)=\delta_{R}\left(U_{\text{w}}\left(z,t\right)\right)=\sum_{{\text{y}}_{s}\left(t\right)\propto \text{w}}q_{s}\left(t\right)$$

Following this, the usability operator δ*_A_* can be defined. On the basis of the reliability of the entire SCA instance, *i.e.*, *R*(*t*) calculated by Equation (41), the usability *E_A_*(*t*) of the entire SCA instance can be calculated by the following formula:

(42) $$E_{A}(t)=\sum_{s=1}^{M_{s\text{y}s}}q_{s}\cdot \delta_{R}(U_{\text{w}}(z,t))=\sum_{s=1}^{M_{s\text{y}s}}\frac{q_{s}(t)}{\sum_{{\text{y}}_{s}(t)\propto \text{w}}q_{s}(t)}(\sum_{y_{s}(t)\propto \text{w}}q_{s}(t))=\sum_{s=1}^{M_{\text{sys}}}\frac{q_{s}(t)}{\sum_{y_{s}(t)\propto \text{w}}q_{s}(t)}R(t)$$

The total output-performance of the entire SCA instance includes both the ones that meet the constraint ***w*** and ones that don't meet the constraint ***w***. The total output-performance operator δ*_G_* is defined as follows. The total output-performance of the entire SCA instance can be calculated by the following formula:

(43) $$E_{G}(t)=\delta_{G}(U(z,t))=\delta_{G}\left(\sum_{s=1}^{M_{s\text{y}s}}q_{s}\left(t\right)z^{{\text{y}}_{s}\left(t\right)}\right)=\frac{dU}{dz}(1)=\sum_{s=1}^{M_{\text{sys}}}q_{s}(t)\cdot y_{s}(t)$$

Thereby, the total output of a certain QoS index of the entire SCA instance can be worked out by the following formula:

(44) $$E(y_{s}(t))=\sum_{s=1}^{M_{\text{sys}}}q_{s}(t)\cdot y_{s}(t)$$

## 6. Numerical Examples

### 6.1. Description for the Example

**Figure 6.**
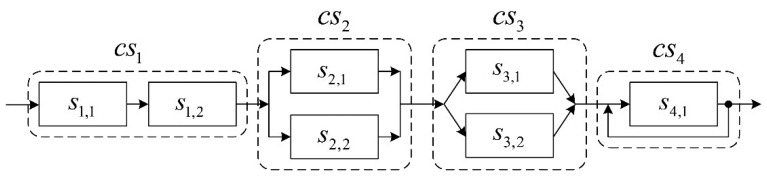
A numerical example.

To describe the calculation and analysis process of multiple QoS indices for the SCA in WSNS, we give a numerical example shown in Figure 6. The given instance of SCA in WSNs is composed of four composite services, *i.e.*, *cs*_1_, *cs*_2_, *cs*_3_, and *cs*_4_. The composite service *cs*_1_ is combined by two atom-services, *i.e.*, *s*_1,1_ and *s*_1,2_, in serial. The composite service *cs*_2_ is composed of two atom-services, *i.e.*, *s*_2,1_ and *s*_2,2_, in parallel. The composite service *cs*_3_ is composed of two atom-services, *i.e.*, *s*_3,1_ and *s*_3,2_, in branch connection with selective probability *p_s_*_3,1_=0.4 and *p_s_*_3,2_=0.6, respectively. The composite service *cs*_4_ is composed by just an atom-service, *i.e.*, *s*_4,1_, that will be executed circularly *c_s_*_4,1_=2 times.

In this numerical example, we pay attention to five types of QoS indices, *i.e.*, reliability, cost, security, execution time, and credibility. The values of five QoS indices of each atom-service in the numerical example are shown in Table 1. In order to express the dynamics and randomness of SCAs in WSNs, the numerical values in numerical examples are selected randomly within the bounds of possibility. In order to investigate the contribution on the total QoS indices by each atom-service, we use percent improvement of each atom-service to find the key atom-services by comparing the improvements of the total QoS indices resulting from them. This can help designers further optimize the total comprehensive QoS by modifying SNs allocation for atom-services, such as allocating more computing resource to those key atom-services.

### 6.2. Calculating the VUGF of Each Atom-Service

According to Formula (11), the VUGF of each atom-service can be obtained as follows:

*s*_1,1_: *U_s_*_1,1_(*z*)=0.98*z*^(5,0.95,16,0.9)^+0.02*z*^(5,0.95,∞,0.9)^, *s*_1,2_: *U_s_*_1,2_(*z*)=0.96*z*^(15,0.9,25,0.85)^+0.04*z*^(15,0.9,∞,0.85)^
*s*_2,1_: *U_s_*_2,1_(*z*)=0.99*z*^(10,0.85,30,0.8)^+0.01*z*^(10,0.85,∞,0.8)^, *s*_2,2_: *U_s_*_2,2_(*z*)=0.97*z*^(20,0.9,25,0.95)^+0.03*z*^(20,0.9,∞,0.95)^
*s*_3,1_: *U_s_*_3,1_(*z*)=0.95*z*^(18,0.95,40,095)^+0.01*z*^(18,0.95,∞,0.95)^, *s*_3,2_: *U_s_*_3,2_(*z*)=0.97*z*^(6,0.85,35,0.8)^+0.03*z*^(6,0.85,∞,0.8)^
*s*_4,1_: *U_s_*_4,1_(*z*)=0.98*z*^(20,0.9,20,0.95)^+0.02*z*^(20,0.9,∞,0.95)^

### 6.3. Calculating the VUGF of Each Composite Service

According to Equation (13), the VUGF of the composite services *cs*_1_, *cs*_2_, and *cs*_4_ can be calculated respectively using the composite operator Ω as follows:

$$\begin{array}{c} \begin{array}{cc} U_{cs1}\left(z\right)=\sum_{l=1}^{M_{s_{1,1}}}\sum_{k=1}^{M_{s_{1,2}}}p_{l}^{s_{1,1}}\cdot p_{k}^{s_{1,2}}\cdot z^{{\text{f}}_{cs1}\left({\text{h}}_{l}^{s_{1,1}},{\text{h}}_{k}^{s_{1,2}}\right)} & U_{cs2}\left(z\right)=\sum_{l=1}^{M_{s_{2,1}}}\sum_{k=1}^{M_{s_{2,2}}}p_{l}^{s_{2,1}}\cdot p_{k}^{s_{2,2}}\cdot z^{{\text{f}}_{cs2}\left({\text{h}}_{l}^{s_{2,1}},{\text{h}}_{k}^{s_{2,2}}\right)} \end{array}\\ U_{cs4}\left(z\right)=\sum_{l=1}^{M_{s_{4,1}}}\sum_{k=1}^{M_{s_{4,1}}}p_{l}^{s_{4,1}}\cdot p_{k}^{s_{4,1}}\cdot z^{{\text{f}}_{cs4}\left({\text{h}}_{l}^{s_{4,1}},{\text{h}}_{k}^{s_{4,1}}\right)} \end{array}$$

The vector functions in the above four expressions, *i.e.*, ${\text{f}}_{cs1}({\text{h}}_{l}^{s_{1,1}},{\text{h}}_{k}^{s_{1,2}})$, ${\text{f}}_{cs2}({\text{h}}_{l}^{s_{2,1}},{\text{h}}_{k}^{s_{2,2}})$, ${\text{f}}_{cs3}({\text{h}}_{l}^{s_{3,1}},{\text{h}}_{k}^{s_{3,2}})$, and ${\text{f}}_{cs4}({\text{h}}_{l}^{s_{4,1}},{\text{h}}_{k}^{s_{4,1}})$ can be obtained by using the suitable operators, which are defined in Equations (15)–(18), according to the types of composite structures as well as the types of QoS indices as follows:

$$U_{cs1}\left(z\right)=0.9408z^{\left(20,0.855,41,0.85\right)}+0.0592z^{\left(20,0.855,\infty ,0.85\right)}$$

$$U_{cs2}\left(z\right)=0.9603z^{\left(30,0.85,30,0.8\right)}+0.0397z^{\left(30,0.85,\infty ,0.8\right)}$$

$$U_{cs3}\left(z\right)=0.38z^{\left(18,0.95,40,0.95\right)}+0.02z^{\left(18,0.95,\infty ,0.95\right)}+0.582z^{\left(6,0.85,35,0.8\right)}+0.018z^{\left(6,0.85,\infty ,0.8\right)}$$

$$U_{cs4}\left(z\right)=0.9604z^{\left(40,0.81,40,0.95\right)}+0.0396z^{\left(40,0.81,\infty ,0.95\right)}$$

### 6.4. Calculating the VUGF of the Entire SCA Instance

The given SCA instance is composed by the four composite services, *i.e.*, *cs*_1_, *cs*_2_, *cs*_3_, and *cs*_4_, in serial. Each of the above composite services can be viewed as a complex Web service concealing its internal structures. Thereby, the VUGF of the entire SCA instance can be obtained through the calculation process like the VUGF of the *cs*_1_ as follows:

$$\begin{array}{ll} U(z) & =\Omega (U_{cs1}(z),U_{cs2}(z),U_{cs3}(z),U_{cs4}(z))\\ & =\sum_{l=1}^{M_{cs1}}\sum_{k=1}^{M_{cs2}}\sum_{i=1}^{M_{cs3}}\sum_{j=1}^{M_{cs4}}p_{l}^{cs1}\cdot p_{k}^{cs2}\cdot p_{i}^{cs3}\cdot p_{j}^{cs4}\cdot z^{\text{f}\left({\text{h}}_{l}^{cs1},{\text{h}}_{k}^{cs2},{\text{h}}_{i}^{cs3},{\text{h}}_{j}^{cs4}\right)}\\ & =0.3297z^{\left(108,0.5593,151,0.8\right)}+0.1533z^{\left(108,0.5593,\infty ,0.8\right)}+0.5050z^{\left(96,0.5004,146,0.8\right)}+0.0950z^{\left(96,0.5004,\infty ,0.8\right)} \end{array}$$

### 6.5. Analysis of QoS Indices with Security Constraint in the VUGF of the Entire SCA

According to the different SCAs and their different application scenarios in WSNs, different users or designers can have different QoS requirements. These different QoS requirements are represented by some QoS constraints. The vector ***w*** = {ω*_cost_*, ω*_security_*, ω*_exec_time_*, ω*_credibility_*} represents the QoS constraints of the entire SCA instance in this example from the users or designers. It is assumed that there are four QoS constraints, including security constraint, from four different types of users:

$$\begin{array}{c} w_{1}=(\omega_{\text{cost}}\leq 96,\omega_{\text{secutity}}\geq 0.5,\omega_{\text{exec}\_\text{time}}\leq 150,\omega_{\text{credibility}}\geq 0.8)\\ w_{2}=(\omega_{\text{cost}}\leq 150,\omega_{\text{secutity}}\geq 0.55,\omega_{\text{exec}\_\text{time}}\leq 200,\omega_{\text{credibility}}\geq 0.8)\\ w_{3}=(\omega_{\text{cost}}\leq 150,\omega_{\text{secutity}}\geq 0.5,\omega_{\text{exec}\_\text{time}}\leq 200,\omega_{\text{credibility}}\geq 0.8)\\ w_{4}=(\omega_{\text{cost}}\leq 95,\omega_{\text{secutity}}\geq 0.55,\omega_{\text{exec}\_\text{time}}\leq 150,\omega_{\text{credibility}}\geq 0.8) \end{array}$$

For the different QoS constraints, the analytical values of QoS indices based on the VUGF of the entire SCA are shown in the corresponding columns of Table 2. The last column of Table 2 shows the analytical values of QoS indices in the case of non-constraint.

The analysis process of QoS indices for the above four different types of constrains are described one by one as follows:

(1) Analysis of QoS indices for the first type of users
  For the first type of users, according to Equations (39) and (40) there is only the third term which satisfies the constraint ***w***_1_, *i.e.*:
  $$U_{{\text{w}}_{1}}\left(z\right)=\sum_{s=1}^{M_{s\text{y}s}}\delta_{{\text{w}}_{1}}\left(q_{s}z^{y_{s}},{\text{w}}_{1}\right)=0.5050z^{\left(96,0.5004,146,0.8\right)}$$
  Thereby, the QoS indices, which the SCA instance can provide to the first type of user, are *cost* = 96, *security* = 0.5004, *exec_time* = 146, and *credibility* = 0.8 respectively. The reliability, which the SCA instance can provide to the first type of user, can obtained according to Equation (41), *i.e.*:
  $$R(t)=\delta_{R}(U_{{\text{w}}_{1}}(z))=\sum_{{\text{y}}_{s}\propto {\text{w}}_{1}}q_{s}=0.5050$$
  The usability provided to the first type of user can be obtained according to Formula (42), *i.e.*:
  $$E_{A}=\sum_{s=1}^{M_{\text{sys}}}q_{s}\cdot R(t)=0.5050\times 0.5050\approx 0.2550$$
(2) Analysis of QoS indices for the second type of users
  Similar to the above analysis process, for the second type of users, there is only the first term which satisfies the constraint ***w***_2_, *i.e.*:
  $$U_{{\text{w}}_{2}}\left(z\right)=\sum_{s=1}^{M_{s\text{y}s}}\delta_{{\text{w}}_{2}}\left(q_{s}z^{y_{s}},{\text{w}}_{2}\right)=0.3297z^{\left(108,0.5593,192,0.8\right)}$$
  Thereby, the QoS indices provided to the second type of user are *cost* = 108, *security* = 0.5593, *exec_time* = 192, *credibility* = 0.8, *reliability* = 0.3297, and *usability* ≈ 0.1087, respectively.
(3) Analysis of QoS indices for the third type of users
  For the third type of users, there are two terms, *i.e.*, the first one and the third one, which satisfy the constraint ***w***_3_, *i.e.*:
  $$U_{{\text{w}}_{3}}\left(z\right)=\sum_{s=1}^{M_{s\text{y}s}}\delta_{{\text{w}}_{3}}\left(q_{s}z^{y_{s}},{\text{w}}_{3}\right)=0.3297z^{\left(108,0.5593,192,0.8\right)}+0.5050z^{\left(96,0.5004,146,0.8\right)}$$
  Thereby, the QoS indices provided to the third type of users are *cost* = 100.74, *security* = 0.5237, *exec_time* = 164.1696, *credibility* = 0.8, *reliability* = 0.8347, and *usability* = 0.6967, respectively.
  Because all the items, whose execution times are less than ∞, are considered in this case, the result of execution time in *U_w_*_3_(*z*) describes the performance of the entire SCA instance. The output of the entire SCA instance can be worked out by the differentiation of this execution time. Thus, we can compare and judge different designs of composite service by evaluating the performance of the corresponding SCA instance, as well as the credibility, the reliability, the usability and so on.
(4) Analysis of QoS indices for the fourth type of users
  For the fourth type of users, there no a term that satisfy the constraint ***w***_4_. Thereby, the SCA instance cannot provide the QoS indices that meet the requirements of the fourth type of user. Assume that the SCA instance has no failures during its execution. Some users are maybe not sensitive to the service execution time. In this case, the total output-performance of the entire SCA instance includes both the ones that meet the constraint ***w*** and ones that don't meet the constraint ***w***. Without considering the execution time constraint, the total expected output-performance provided to users can be obtained according to Formula (44), *i.e.*, *cost* = 109.7640, *security* = 0.5704, and *credibility* = 0.8.

### 6.6. Performance Sensitivity Analysis and Performance Optimization Subjected to the Security Constraint of the Entire SCA

**Figure 7.**
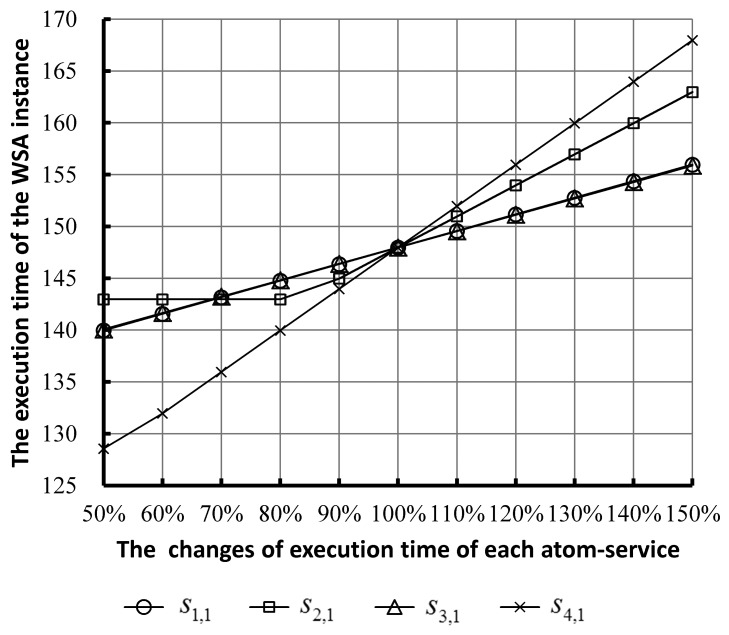
The influence on the execution time of the SCA instance by the changes of the execution time of each atom-service.

For designers and managers of an SCA instance, it is very important and valuable to know the degree of influence on the performance of an SCA instance resulting from the performance changes of each atom-service subjected to the security constraint of the entire SCA. The performance of an SCA instance can be represented by its execution time. We chose four atom-services, *i.e.*, *s*_1,1_, *s*_2,1_, *s*_3,1_ and *s*_4,1_, in each composite service in the SCA instance to study their performance sensitivities. In order to analyze the influence on the total execution time by each atom-service, a set of experiments have been performed. Figure 7 shows the variation of the execution time of the instance caused by the changes of the execution time of each atom-service.

The horizontal axis shows that the execution times of each atom-service gradually increase from 50% to 150% on the basis of the original values shown in Table 1. The execution time of the SCA instance, represented by these curves in Figure 7, gradually increases as the execution times of each atom-service increase.

**Figure 8.**
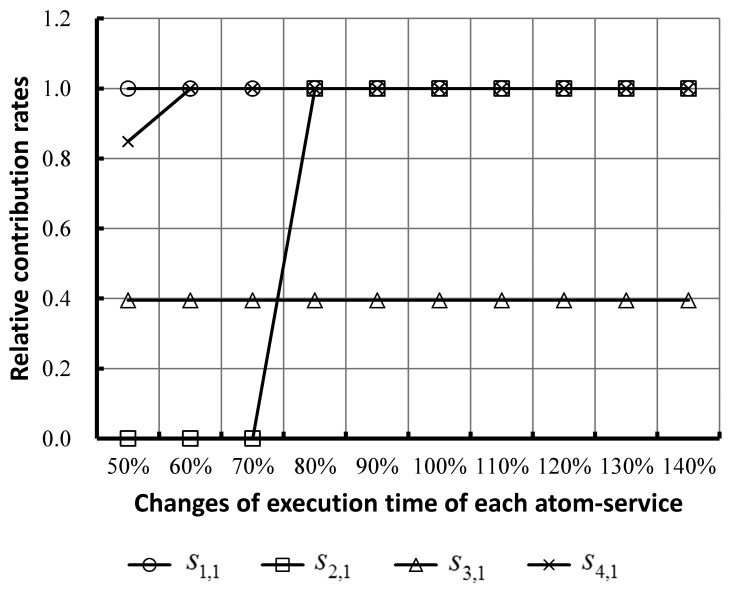
Relative contribution rates on total execution time by each atom-service.

On this basis, we analyzed the contribution on the total execution time by each atom-service. Figure 8 shows the variation of relative contribution rates on total execution time with the changes of execution time of each service. The relative contribution rates equal the growth rate of total execution time per incremental execution time of each service.

**Figure 9.**
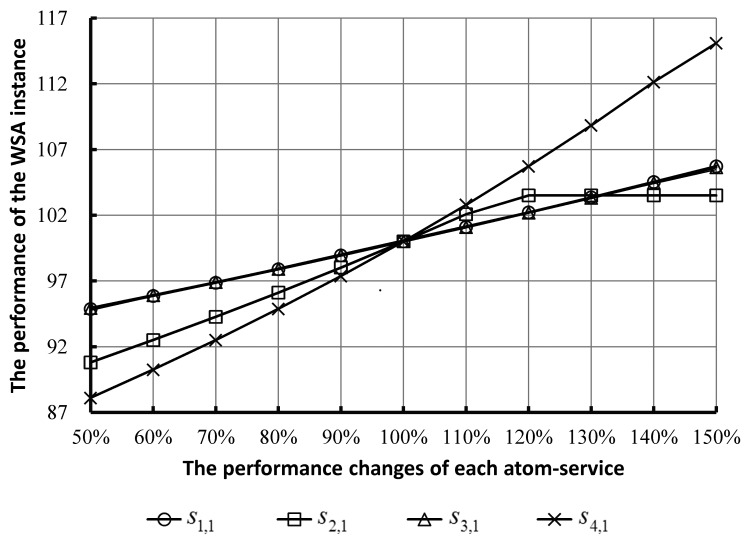
The performance influence on the SCA instance by the performance changes of each atom-service.

In order to analyze the performance influence on the SCA instance caused by performance changes of each atom-service, we studied the performance variation of the SCA instance caused by the performance changes of each atom-service. Figure 9 shows the gradual improvement of performance of the SCA instance as the performances of each atom-service increase from original ones of 50% to 150%.

**Figure 10.**
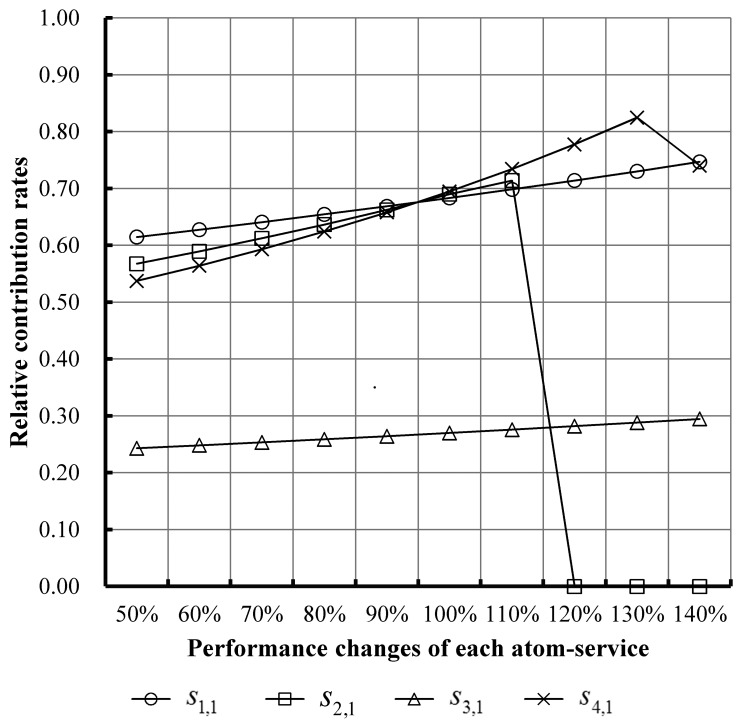
Relative contribution rates on total performance of the SCA instance by each atom-service.

On this basis, we analyzed the contribution on total execution time by each atom-service. Figure 10 shows the variation of relative contribution rates on total performance with the changes of performance of each atom-service. The relative contribution rates equal the growth rate of total performance per incremental performance of each atom-service.

From Figures 9 and 10, we draw the following conclusions:

(1) These curves in Figure 9 show a rising trend from left to right in the mass, which indicates that the performance changes of each atom-service can influence the performance of the SCA instance. With the increase (decrease) of the performance of each atom-service, the performance of the SCA instance increases (decreases) gradually.
(2) The gradients of these curves are not all the same. This indicates the degrees of influence caused by each atom-service are not all the same on the total performance of the SCA instance. From Figure 10, one can see the similar situation that the contributions on the total performance by each atom-service are not same. It can be seen that the gradient of the curve corresponding to *s*_4,1_ is the largest, which indicates the degree of influence on the total performance of the SCA instance caused by *s*_4,1_ is the largest. The gradient of the curve corresponding to *s*_3,1_ is the smallest. In the other words, the contribution rate of *s*_4,1_ on the total performance of the SCA instance is the largest. On the contrary, that of *s*_3,1_ is the smallest.
(3) Thus, the designers and managers of the SCA instances can adjust the deployment of atom-services within an SCA instance to optimize the total performance according to the contribution rates of each atom-service. Specifically, they can improve the performance of atom-services with the highest or higher contribution rate. There are various approaches to improve the performance of atom-services. For example, in our experiment the management server is deployed on a cloud computing platform. Thus, we can add more CPU or/and more memory into the virtual machines where these atom-services are deployed. In addition, when the computing resource of the host where these atom-services are deployed is limited, we still can emigrate these atom-services to other virtual machines with better performance (such as more CPU or/and more memory).
(4) Sometimes, the total computing resources, *i.e.*, CPU and memory, provided to the SCA instance by the cloud computing platform are limited and cannot meet the demand. In this case, the designers and managers of the SCA instances can allocate relatively more computing resources to those atom-services with higher contribution rates, which can improve the performance of the SCA instance as much as possible.
(5) From the curve corresponding to *s*_2,1_ in Figures 9 and 10, it can be seen that the performance improvement of the SCA instance stops after the performance of *s*_2,1_ grows to the original ones' 120%. Analyzing the composition structure of the SCA instance, we found that the parallel structure connecting *s*_2,1_ and *s*_2,2_ is the primary reason. In the other words, the contribution rate of *s*_2,1_ is limited by the performance of *s*_2,2_. In this case, the designers and managers of the SCA instances should not allocate more computing resource to *s*_2,1_.

Generally, high performance and high security may be contratictory as well as high credibility, high reputation and low cost. The value of the suggested analysis method lies in that they provide the basis for the tradeoff between these QoS indices, including security, by the users or designers.

## 7. Conclusions and Future Work

Services composition is one of the key software development techniques in multi-service WSNs. The QoS of SCAs is confronted with severe challenges due to the open, dynamic, and complex nature of WSNs. The traditional QoS analysis techniques, fox example Boolean Models, Markov Process and Monte-Carlo simulation technique, have some defects such as being too time-consuming, easy to cause state space explosions and unsatisfactory assumptions of component execution independence. In addition, the traditional analysis techniques seldom consider the relationship between the QoS indices. Though the classic UGF technique shows the obvious advantages of speediness and precise on the analysis of QoS indices, its computational efficiency limits the application in large-scale WSNs because only one QoS index can be analyzed simultaneously.

Being different from the general software system, there are huge number and variety of sensor services that may join or leave the system at any time in a WSNs service system. When a WSNs service system achieves a certain scale, using traditional analysis methods may cause a state space explosion, while using the classic UGF the computational efficiency and analyzing ability for comprehensive indices are difficult to meet the requirements. Depending on the abilities of parallel computation and comprehensive analysis, VUGF can successfully deal with the characteristics of large-scale sensor services and high dynamics.

Aiming at the defects in the existing methods, an improved UGF technique—VUGF—is proposed in this paper, by which the multiple QoS indices can be simultaneously analyzed and the computational efficiency is improved obviously. The VUGF eliminates the limitation for component execution independence, and more fits the actual execution of SCAs. In addition, the VUGF has very small consumption of time and space to eliminate the risk of state space explosion.

In the use of VUGF for estimating the multiple QoS indices of an SCA instance, the estimation accuracy is related to the number of divided states. The more divided states, the more accurate the estimation, at the same time more complicated the computation will be. Combining like terms and redistricting states can further reduce the calculation workload.

The suggested comprehensive QoS indices analysis based on the VUGF considers the relationship between multiple QoS indices, including security. It can be used for the evaluation of the comprehensive QoS of SCAs subjected to the security constraint in WSNs. Therefore, it can be effectively applied to the optimal design of multi-service WSNs.

In the future, we are going to study a fast optimization method for multiple QoS indices of SCAs in WSNs based on the hybrid optimization algorithm and the analysis method proposed in this paper. At first, we will further research the multiple QoS indices model of SCAs in WSNs based on the MSS theory. Then, we will further research the comprehensive evaluation method for the multiple QoS indices of SCAs in WSNs based on the VUGF. On this basis, we will research the fast optimization algorithm for multiple QoS indices of SCAs in WSNs based on the hybrid optimization algorithm.

## Figures and Tables

**Table 1.** The QoS indices values of each service in the numerical example.

**QoS Indices**	***cs*_1_**		***cs*_2_**		***cs*_3_**		***cs*_4_**
			
	***s*_1,1_**	***s*_1,2_**	***s*_2,1_**	***s*_2,2_**	***s*_3,1_**	***s*_3,2_**	***s*_4,1_**
Reliability	0.98	0.96	0.99	0.97	0.95	0.97	0.98
Cost	5	15	10	20	18	6	20
Security	0.95	0.90	0.85	0.90	0.95	0.85	0.90
Execution Time	16	25	30	25	40	35	20
Credibility	0.90	0.85	0.80	0.95	0.95	0.80	0.95

**Table 2.** The analytical values of QoS indices based on the VUGF of the entire SCA.

**QoS Indices**	**QoS Constraints**				

	**ω_1_**	**ω_2_**	**ω_3_**	**ω_4_**	**φ**
Reliability	0.5050	0.3297	0.8347	-	-
Cost	96.00	108.00	100.74	-	109.76
Security	0.5004	0.5593	0.5237	-	0.5704
Execution Time	146	192	164	-	-
Credibility	0.8	0.8	0.8	-	0.8
Usability	0.2550	0.1087	0.6967	-	-
